# Mechanisms utilized by feline adipose-derived mesenchymal stem cells to inhibit T lymphocyte proliferation

**DOI:** 10.1186/s13287-019-1300-3

**Published:** 2019-06-25

**Authors:** Nopmanee Taechangam, Smita S. Iyer, Naomi J. Walker, Boaz Arzi, Dori L. Borjesson

**Affiliations:** 1 Department of Pathology, Microbiology and Immunology, Vet Med 3A, University of California, 1285 Veterinary Medicine Mall, Davis, CA 95616 USA; 2 Department of Surgical and Radiological Sciences, University of California, Davis, CA 95616 USA; 3 Veterinary Institute for Regenerative Cures, School of Veterinary Medicine, University of California, Davis, CA 95616 USA

**Keywords:** Mesenchymal stem cell, Adipose tissue, Feline, Immunomodulation, soluble mediators, ligands

## Abstract

**Background:**

Feline adipose-derived mesenchymal stem cells (ASCs) have been successfully used in clinical trials for the treatment of immune-mediated diseases with T cell dysregulation. However, the immunomodulatory pathways utilized by feline ASCs to suppress T cell activation have not been fully determined. We investigated the mechanisms used by feline ASCs to inhibit T cell proliferation, including the soluble factors and the cell-cell contact ligands responsible for ASC-T cell interaction.

**Methods:**

The immunomodulatory activity of feline ASCs was evaluated via cell cycle analysis and in vitro mixed leukocyte reaction using specific immunomodulatory inhibitors. Cell-cell interactions were assessed with static adhesion assays, also with inhibitors.

**Results:**

Feline ASCs decrease T cell proliferation by causing cell cycle arrest in G0–G1. Blocking prostaglandin (PGE_2_), but not IDO, partially restored lymphocyte proliferation. Although PDL-1 and CD137L are both expressed on activated feline ASCs, only the interaction of intercellular adhesion molecule 1 (ICAM-1, CD54) with its ligand, lymphocyte function-associated antigen 1 (LFA-1, CD11a/CD18), was responsible for ASC-T cell adhesion. Blocking this interaction reduced cell-cell adhesion and mediator (IFN-γ) secretion and signaling.

**Conclusions:**

Feline ASCs utilize PGE_2_ and ICAM-1/LFA-1 ligand interaction to inhibit T cell proliferation with a resultant cell cycle arrest in G0–G1. These data further elucidate the mechanisms by which feline ASCs interact with T cells, help define appropriate T cell-mediated disease targets in cats that may be amenable to ASC therapy, and may also inform potential translational models for human diseases.

**Electronic supplementary material:**

The online version of this article (10.1186/s13287-019-1300-3) contains supplementary material, which is available to authorized users.

## Background

Mesenchymal stem cells (MSCs) are a heterogeneous, multipotent stromal cell population, capable of proliferating in vitro as plastic-adherent cells with fibroblast-like morphology and differentiating into bone, cartilage, and adipose cells [1]. Aside from their regenerative properties, MSCs also possess immunomodulatory properties and have been used extensively to treat a wide variety of immune-mediated diseases, both in human and veterinary medicine [2]. In veterinary medicine, adipose-derived mesenchymal stem cells (ASCs) have been successfully used to treat cats with feline chronic gingivostomatitis (FCGS), a severe debilitating oral immune-mediated disease [3, 4], but the mechanisms by which feline ASCs modulate the immune system have not been fully elucidated.

MSCs can be immunosuppressive and can inhibit the mitogen-induced response of naïve T lymphocytes [5], both CD4+ and CD8+, as well as of natural killer (NK) cells [6]. There are several possible mechanisms by which MSCs may inhibit T lymphocyte proliferation including the induction of apoptosis, cell cycle arrest, induction of a phenotype switch to regulatory T cells, or decreasing T lymphocyte activation, ultimately leading to anergy [7]. Suppression of T cell proliferation by MSCs can be mediated by secreted soluble factors because the separation of MSCs and activated T lymphocytes by a transwell can inhibit proliferation without the presence of cell-cell contact [8, 9]. However, direct cell-cell contact is also important in MSC regulation of T lymphocyte function in both humans and cats [8, 10].

MSC immunosuppressive functions require preliminary activation by immune cells through the secretion of IFN-γ, a pro-inflammatory cytokine [6, 11]. Activated feline ASCs secrete high levels of immunomodulatory mediators, including indoleamine 2,3 dioxygenase (IDO), prostaglandin E2 (PGE_2_), interleukin (IL)-6, IL-8, and transforming growth factor beta (TGFβ) similar to human MSCs. However, unlike human MSCs, the secretion of PGE_2_ and IDO by feline ASCs is significantly reduced in the absence of direct-cell contact [8]. In contrast to other species, including humans, dogs, and horses, feline ASCs inhibit lymphocyte proliferation in the context of significantly increased concentration of IFN-γ [8, 12, 13].

Several cell ligand pairs have been implicated in MSC and T lymphocyte adhesion and signaling that subsequently impact the secretion of soluble immunomodulatory factors. These ligand pairs include ICAM-1/LFA-1, VCAM-1/VLA-4, and PDL-1/PD-1 [14, 15]. In murine MSCs, ICAM-1 is a requirement for lymphocyte–MSC adhesion and blocking ICAM-1 ligand reduced T cell accumulation around MSCs and reversed the suppression of lymphocyte proliferation [15]. ICAM-1 also plays a crucial role, particularly in T cell interactions with antigen-presenting cells, and is essential for the immunosuppressive effects of murine bone marrow-derived MSCs [15, 16]. PDL1 and PDL-1, a negative costimulatory molecule, has also been implicated in contact-dependent suppression in human MSCs [14, 17].

Our data from in vivo studies suggest that one mechanism by which feline ASCs decrease T cell-mediated inflammation is via the induction of CD8+ regulatory T cells [4]. Other groups have shown that CD137-CD137L co-stimulation can induce CD8+ regulatory T cells in the presence of IFN-γ [8, 18, 19]. Co-stimulation through the CD137-137L pathway also enhances suppressive T cell function and induces activated T cell anergy in human immune-mediated diseases [20, 21].

The purpose of this study was to define the mechanisms used by feline ASCs to suppress T lymphocyte proliferation, focusing on both soluble mediators and direct cell-cell contact ligands. Similar to human ASCs, we found that (1) feline ASCs induce G0–G1 cell cycle arrest in mitogen-activated T lymphocytes, (2) PGE_2_ is a primary soluble factor partially responsible for the inhibition of T lymphocyte proliferation, and (3) a crucial ligand pair mediating feline ASC and T lymphocyte adhesion and secretion profile is ICAM-1/LFA-1. Notably, the increase in IFN-γ secretion induced after feline ASC-T cell direct interaction is abrogated when ICAM-1 is blocked. These findings shed light on both shared and unique aspects of feline ASC biology that may underscore how the administration of ASCs results in long-term reprograming of the immune system in cats with FCGS.

## Materials and methods

### Feline adipose-derived mesenchymal stem cells (ASCs)

Low passage (P1–P5) adipose-derived feline mesenchymal stem cells (ASCs) were isolated from subcutaneous feline adipose tissue surgically obtained from specific pathogen-free (SPF) cats or from client-owned cats undergoing routine surgery. Fat collection was conducted according to a protocol approved by the Institutional Animal Care and Use Committee, and the Clinical Trials Review Board, UCD (protocol number 18422). All owners of client-owned cats signed an informed consent form. All cats were free of feline immune deficiency virus and feline leukemia virus infection. ASC isolation and expansion was performed at the UC Davis William R. Pritchard Veterinary Medical Teaching Hospital Regenerative Medicine Laboratory, exactly as previously described [4].

### ASC culture and expansion

Feline ASCs were expanded as previously described [22]. In brief, cryopreserved ASCs were thawed in a 37 °C water bath and seeded into tissue culture flasks with Dulbecco’s modified Eagle’s medium (DMEM; Corning Life Sciences), 10% fetal bovine serum (HyClone Inc.), and 1% penicillin/streptomycin (Thermo Fisher Scientific) and incubated at 37 °C in 5% CO_2_ at 90% humidity. Feline ASCs from passages 2–5 were used in the experiments. All ASC lines passed quality control assays including bacterial culture (all were sterile), high viability (> 90%), positive for CD90 (identity marker), negative for CD18 (purity marker), and negative for endotoxin and Mycoplasma.

### Peripheral blood monocular cell (PBMC) inhibition assay—mixed leukocyte reaction (MLR)

Feline ASCs were tested for their capability to inhibit lymphocyte proliferation with a mixed leukocyte reaction (MLR), carried out as previously described [4]. In brief, PBMCs were isolated from whole blood using gradient centrifugation and were co-incubated with irradiated ASCs in culture wells at a 1:5 (PBMC to ASC) ratio and activated with 5 mg/mL concanavalin A (ConA; Sigma-Aldrich). Cells were co-cultured for 4 days. Control wells included PBMCs alone and ConA-stimulated PBMCs. To determine indoleamine 2,3-dioxygenase (IDO) activity, the experiment was run as described; however, the media was supplemented with l-tryptophan (Sigma-Aldrich) to a final concentration of 600 μM. To measure proliferation, wells were spiked with 5-bromo-29-deoxyuridine (BrdU) at day 3 and then cells were collected and processed per manufacturer’s instructions (BrdU Flow Kit; BD Biosciences) at day 4.

Some protocols included the addition of antibodies to block TGF-β (10 μg/mL, anti TGF-β1 mouse monoclonal IgG, clone 9106, R&D systems), or interferon gamma (IFN-γ, 15 μg/mL, goat anti-feline polyclonal antibody #AF674, R&D systems), or chemicals to block prostaglandin E2 (PGE_2_, 10 μm/mL, indomethacin, Cayman Chemical), or IDO (500 μM/mL 1-methyl-l-tryptophan (Sigma-Aldrich), a competitive inhibitor of tryptophan). Inhibitor concentration was determined by titration studies in our laboratory or based on previous publications [23].

### Feline ASCs and PBMC phenotyping

For the analysis of surface expression on feline ASCs and PBMCs, cells were harvested and resuspended at a concentration of 1 × 10^6^ cell/mL in flow buffer (DPBS, 1% normal equine serum, 10 mM EDTA, and 0.1% sodium azide). Cells were incubated with antibodies for 30 min at room temperature. Antibodies included mouse anti-feline CD4-PE (clone 3-4F4, Southern Biotech), mouse anti-feline CD5-FITC (clone f43, Southern Biotech), mouse anti-feline CD8α-PE (clone Fe1.10E9, Leukocyte Antigen Biology Laboratory, UC Davis), mouse anti-human I-CAM 1 (CD54, clone MEM-111, Thermo Fisher Scientific), rat anti-mouse CD137L (clone TKS-1, Bio X Cell), polyclonal goat anti-human-PD-1 (cat#AF1086, R&D systems), polyclonal goat anti-human-PDL-1/B7-H1 (cat#AF156, R&D systems). The secondary antibody used for indirect labeling was R-Phycoerythrin F(ab’)_2_ Fragment donkey anti-goat IgG (Jackson ImmunoResearch Inc.) and Fluorescein Rabbit Anti-Rat IgG (Vector Laboratories). Cells were analyzed with a Beckman-Coulter Cytomics FC500 flow cytometer. Data analyses were done on Flowjo flow cytometry software (Tree Star, Inc.).

### Cell cycle analysis

Lymphocyte DNA content was determined with 7-amino-actinomycin D (7-AAD; BD Biosciences) incorporation to distinguish between lymphocyte populations in the S-phase, G1 phase, and G2–M phase in conjunction with BrdU incorporation (FITC BrdU Flow Kit; BD Biosciences). PBMCs were collected for 4 consecutive days from co-incubation experiments with feline ASCs. Cells were analyzed with a Beckman-Coulter Cytomics FC500 flow cytometer. Data analyses were done on Flowjo flow cytometry software (Tree Star, Inc.).

### Detection of intracellular IFN-γ

PBMCs were collected from the MLR on day 4 and resuspended in RPMI 1640 media with 10% heat-inactivated FBS, 1% Gluta-Max, 1 mM sodium pyruvate, 1% penicillin-streptomycin, 2 mM HEPES, 0.1% MEM NEAA, and 55 μM β-mercaptoethanol. Collected cells were stimulated with 25 ng/mL Phorbol-12-Myristate-13-Acetate (PMA, Sigma-Aldrich) and 500 ng/mL ionomycin (Sigma-Aldrich), treated with 1 μg/mL Brefeldin A and incubated for 3 h at 37 °C. Cells were then washed, stained with a viability dye (Fixable Viability Dye eFlour®780, eBioscience), fixed with 2% paraformaldehyde, permeabilized in wash buffer (DPBS with 0.5% bovine serum albumin, 0.1% saponin, and 0.02% sodium azide), and stained with anti-bovine IFN-γ-AlexaFluor647 antibody (clone CC302, Bio-Rad). Cells were analyzed with Beckman-Coulter Cytomics FC500 flow cytometer. Data analyses were done on Flowjo flow cytometry software (Tree Star, Inc.).

### Detection of secreted mediators

IDO, nitric oxide (NO), PGE_2_, and IFN-γ were measured in the MLR culture supernatant collected on day 4. Supernatants were stored at − 80 °C until analyzed. PGE_2_ and IFN-γ concentration were measured using commercially available feline-specific ELISA kits (Enzo Life Sciences and R&D systems, respectively), according to the manufacturer’s instructions [8]. IDO activity was determined through the measurement of colorimetric kynurenine level assay, and NO was measured with a Griess reagent system (Griess Reagent System, Promega Corporation), both performed exactly as previously described [12]. All samples were read on a Synergy HT Multi-Mode microplate reader with Gen5 software (Biotek).

### MSC-PBMC static adhesion assay

Static adhesion assay was modified from Ren et al. [15]. In brief, feline ASCs were plated in 24-well plates (5 × 10^4^ cells/well) in 750 μL standard culture medium. Isolated PBMCs were fluorescently labeled with CellTracker™ Green CMFDA (5-chloromethylfluorescein diacetate, Thermo Fisher Scientific) and activated with 5 μg ConA for 1 h prior to adding to the ASCs (1 × 10^6^ cells/well). Cells were permitted to adhere to ASCs for 2 h at 37 °C in 5% CO_2_. The plates were then rotated at 300 rpm for 5 min and washed with DPBS twice to remove non-attached PBMCs. Fluorescence was detected using a Synergy HT Multi-Mode microplate reader at 485-nm wavelength prior to and after washing to quantify the change in fluorescent intensity. The plate was also visualized and photographed on an inverted fluorescent microscope (EVOS FL, Thermo Fisher Scientific). In some experiments, blocking antibodies to ICAM 1 (anti-human CD54, clone MEM-111, Thermo Fisher Scientific), LFA-1 (anti-human clone R7.1, eBioscience), CD137 (anti-mouse clone 17B5, Bio X Cell), CD137L (anti-mouse clone TKS-1, Bio X Cell), PD-1 (anti-human PD-1, polyclonal goat IgG, R&D systems), or PDL-1 (anti-human PDL-1, polyclonal goat IgG, R&D systems) were added to determine which ligands mediated PBMC-ASC adhesion. The concentration of antibodies used was determined by titration studies in our laboratory.

### Statistical analyses

Data analysis was performed using GraphPad Prism version 7 software (GraphPad Software). All experiments were performed with *n* = 5 (feline ASC lines and PBMCs donors) unless otherwise indicated. Statistical significance between two groups was determined by non-parametric Mann-Whitney-Wilcoxon test due to small sample size. *p* values < 0.05 were considered statistically significant.

## Results

### Activated feline CD4+ and CD8+ T lymphocytes both secrete IFN-γ

Feline ASCs decrease activated T cell proliferation and secretion of pro-inflammatory cytokines, notably tumor necrosis factor alpha (TNF-α). However, unlike other species, including people, dogs, and horses, feline ASCs inhibit lymphocyte proliferation in the presence of increased IFN-γ concentration when ASCs are in direct contact with lymphocytes [6, 8, 12, 13, 24]. We previously hypothesized that feline ASCs could be licensed by IFN-γ and this signaling may be critical for the long-term reprograming of CD8+ regulatory T lymphocytes [25–27]. Our previous work did not identify the cell types responsible for IFN-γ secretion in our assays. As ASCs inhibit lymphocyte proliferation regardless of cell-cell contact, high IFN-γ concentration can be used as a surrogate marker of contact-mediated T cell inhibition and the reduction of IFN-γ secretion can be used as a marker of effective blockade of this pathway.

**Fig. 1:**
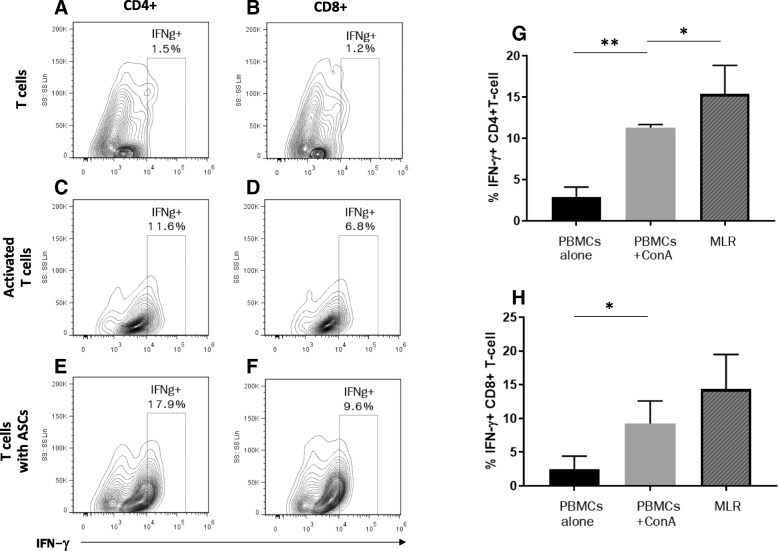
Both activated feline CD4 and CD8+ T cells secrete IFN-γ. Intracellular IFN-γ + cell population in **a** unstimulated CD4+ cells, **b** unstimulated CD8+ cells, **c** CD4+ cells stimulated with ConA, **d** CD8+ cells stimulated with ConA, **e** CD4+ cells in co-incubation with feline ASCs, and **f** CD8+ cells in co-incubation with feline ASCs. **g** Percentage of IFN-γ + CD4+ T cell increased after mitogen activation (*p* = 0.008) and was further augmented with feline ASC co-incubation (*p* = 0.02) **h** Percentage of IFN-γ + CD8+ T cell increased after mitogen activation (*p* = 0.02) with a trend to increase with feline ASC co-incubation, but was not statistically significant. Representative flow cytometric images and data from 5 independent experiments. *p < 0.05

We found that feline CD4 and CD8 T lymphocytes both secrete IFN-γ after mitogen activation (Fig. 1a–d) and the secretion of IFN-γ from CD4+ T lymphocytes is significantly increased upon co-incubation with feline ASCs (*p* = 0.02; Fig. 1g), and the level of IFN-γ is sustained with a tendency to increase in CD8+ T lymphocytes in the presence of feline ASCs (Fig. 1h).

### Feline ASCs decrease activated PBMC viability and inhibit lymphocyte proliferation through the induction of G0–G1 cell cycle arrest

**Fig. 2:**
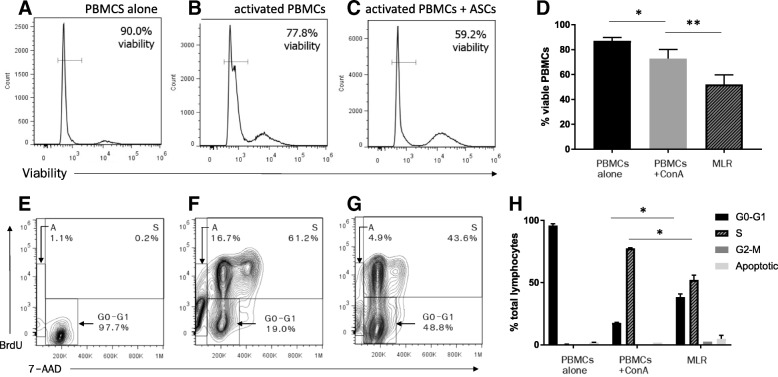
Feline ASCs decrease activated PBMC viability and induce cell cycle arrest in activated T lymphocytes**.** Representative images of flow cytometric analysis on day 4 from 5 MLR experiments with condition of **a** PBMCs only, **b** mitogen-activated PBMCs, and **c** PBMCs in co-incubation with feline ASCs. **d** the percentage of viable PBMCs decreased after mitogen activation (*p* = 0.04) and was further exacerbated by the co-incubation with feline ASCs (*p* = 0.008). Flow cytometric scatter plot of cell cycle analysis on T lymphocyte DNA content (7-AAD) and proliferation determined through BrdU incorporation of **e** PBMCs only, **f** mitogen-activated PBMCs, and **g** PBMCs in co-incubation with feline ASCs. **h** Percentage of T cells in the apoptotic, G0–G1, S, and G2–M phases from cell cycle analysis revealed that the percentage of T lymphocytes in the G0–G1 phase increased with a concurrent decrease in the S-phase upon co-incubation with feline ASCs (*p* = 0.03). *p < 0.05 **p < 0.01

Feline ASCs inhibit mitogen-activated T cell proliferation with and without the presence of cell-to-cell contact [8], but the mechanism of action is not known. Here we demonstrate that feline PBMC viability decreased upon mitogen activation (*p* = 0.04) and was even further exacerbated by the co-incubation with feline ASCs (*p* = 0.008; Fig. 2a–d). Additionally, cell cycle analysis revealed that the percentage of T lymphocytes in the G0–G1 phase increased with a concurrent decrease in the S-phase upon co-incubation with feline ASCs (*p* = 0.03). However, feline ASCs did not undergo increased apoptosis compared to the mitogen-activated condition (Fig. 2d–f). These findings suggest that feline ASCs inhibit activated PBMC viability and inhibit the proliferation of mitogen-activated T lymphocytes through the induction of G0–G1 cell cycle arrest.

### Feline ASC secretion of PGE_2_ is partially responsible for inhibiting lymphocyte proliferation

Our previous data suggested that there are at least 2 mechanisms by which feline ASCs inhibit activated T cell proliferation, one relying on direct contact between the ASCs and T lymphocytes and another dependent on soluble factors [3, 8]. Feline ASCs constitutively produce low concentrations of immunomodulatory mediators in the absence of activation, but secretion is much higher in the presence of mitogen-activated T cells, particularly the secretion of IDO and PGE_2_ which is enhanced by direct cell contact [8]. Although the feline ASC secretion profile has largely been determined [8], we wanted to (1) more fully elucidate mediators secreted by feline ASCs (in the presence and absence of contact) and (2) identify the soluble mediators critical for the inhibition lymphocyte proliferation, focused on TGF-β, IFN-γ, PGE_2_, and IDO.

**Fig. 3:**
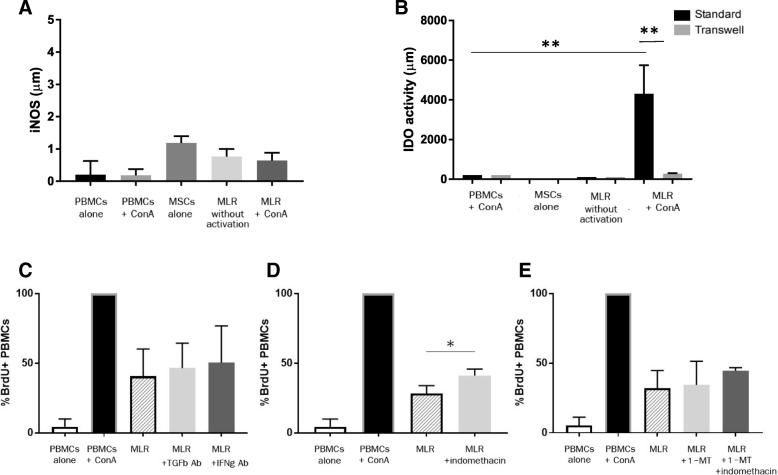
PGE2 secretion partially mediates feline ASC inhibition of T lymphocyte proliferation. **a** Measurement of iNOS production from supernatant of MLR on day 4 of co-incubation. Data from 5 independent experiments. **b** Measurement of IDO production from supernatant of MLR on day 4 of feline ASC and PBMC co-incubation with and without transwell. Data from 5 independent experiments. Degree of proliferation in the MLR with inhibition of soluble mediators: **c** addition of TGF-β and IFN-γ blocking antibody, **d** blocking of PGE2 with indomethacin, **e** blocking IDO with 1-methyltryptophan and PGE2 with indomethacin. Percentage of proliferation is normalized to 100% on mitogen activated condition for comparability. Blocking PGE2 significantly hindered the degree of suppression (*p* = 0.03). *p < 0.05, **p < 0.01

Nitric oxide (NO) may play an important role in human MSC-induced T lymphocyte immunosuppression [28]; however, feline ASCs did not secrete substantial quantities of NO even in the presence of activated T lymphocytes (Fig. 3a). Like PGE_2_, IDO was secreted by activated feline ASCs and PBMCs [8] but only in the presence of direct cell-contact (*p* = 0.008; Fig. 3b).

We found that PGE_2_ was partially responsible for the inhibition of activated lymphocytes by feline ASCs (*p* = 0.03); however, blocking TGF-β, IFN-γ, and IDO did not significantly restore lymphocyte proliferation (Fig. 3c–e). Blocking both PGE_2_ and IDO demonstrated a slight increase, but did not significantly restore lymphocyte proliferation (Fig. 3e).

### ICAM-1 mediates the adhesion between feline ASCs and T lymphocytes

**Fig. 4:**
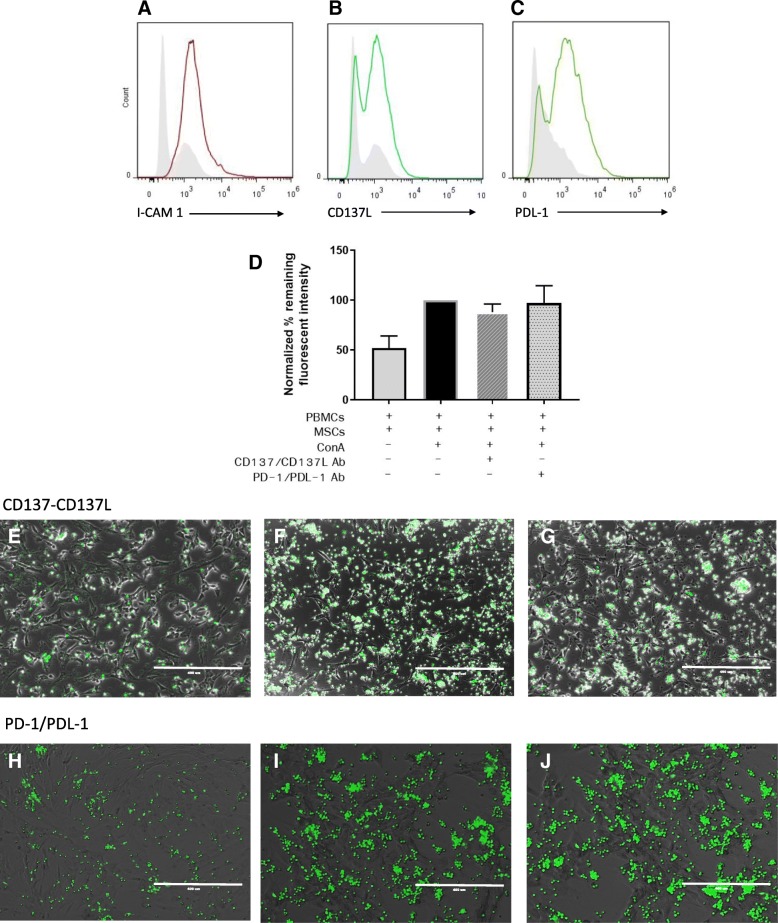
ICAM-1, CD137L, and PDL-1 are all expressed on activated feline ASCs; however, CD137L and PDL-1 do not mediate MSC-T cell adhesion. Expression of **a** ICAM-1, **b** CD137L, and **c** PDL-1 ligands on activated feline ASCs. Gray histogram indicated background fluorescence of unstained samples. **d** Percentage of remaining fluorescence intensity from CMFDA-labeled PBMCs after removal of non-adherent cells from static adhesion assay after the addition of CD137/CD137L and PD-1/PDL-1 blocking antibodies. Data is normalized to 100% on a standard MLR condition for comparability. Fluorescent images of static adhesion assay demonstrating adherent lymphocytes to ASCs from **e**, **h** non-activated MLR, **f**, **i** stimulated MLR with ConA, and **g**, **j** stimulated MLR with ConA and addition of CD137/CD137L and PD-1/PDL-1 blocking antibodies respectively. Scale bar = 400 μm. Representative flow analysis images in **a**–**c** from 3 independent experiments. Data gathered in **d** from 5 independent experiments

Cell-cell contact is an important factor for MSC-mediated T cell immunosuppression [12, 17, 29]. Given the importance of cell-cell contact and the unique contact-dependent mediator secretion profile for feline ASCs in particular, we investigated the potential role of 3 ASC cell surface receptors [CD54 ICAM-1, PDL-1, and CD137L] to regulate T cell-feline ASC adhesion. We first determined if ICAM-1, PDL-1, and CD137L were expressed on feline MSCs and whether co-incubation of activated T cells with MSCs resulted in increased expression of these surface receptors. Flow cytometric analysis revealed that activated feline ASCs expressed ICAM-1, CD137L, and PDL-1 on their surface (Fig. 4a–c). Activated feline T cells express LFA-1 [30], PD-1 [31], and CD137 [unpublished data]; however, it was unknown if, similar to human MSCs, ASC co-incubation with activated T cells would decrease PD-1, CD137, and LFA-1 expression on activated T cells. We found that, unlike human MSCs, feline ASCs did not decrease PD-1 expression on activated T cells (Additional file 1).

**Fig. 5:**
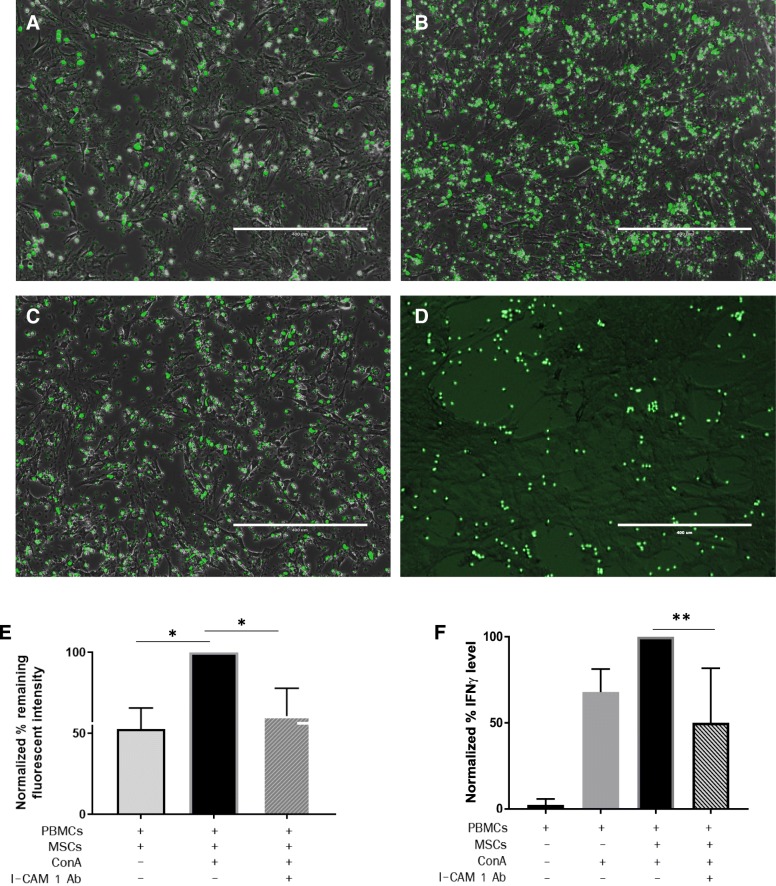
I-CAM 1 ligand mediates the adhesion between feline ASCs and T lymphocytes. Fluorescent images of static adhesion assay demonstrating adherent lymphocytes to feline ASCs from **a** non-activated MLR, **b** stimulated MLR with ConA, **c** stimulated MLR with ConA and addition of ICAM-1 blocking antibodies, and **d** stimulated MLR with ConA and addition of ICAM-1/LFA-1 blocking antibodies. Scale bar = 400 μm. **e** Percentage of remaining fluorescence intensity from CMFDA-labeled PBMCs after removal of non-adherent cells from static adhesion assay with added condition of ICAM-1 blocking antibodies. Data is normalized to 100% on standard MLR condition for comparability. **f** Changes in IFN-γ concentration in the MLR after addition of ICAM-1 blocking antibodies. Data from 5 experiments, normalized to 100% on standard MLR condition for comparability. *p < 0.05, **p < 0.01

We then utilized blocking antibodies against ICAM-1/LFA-1, CD137/CD137L, and PD-1/PDL-1 to directly test whether these ligand pairs mediated feline ASC-lymphocyte adhesion in static conditions. Neither the blockade of CD137/CD137L nor PD-1/PDL-1 significantly altered T cell-ASC adhesion (Fig. 4d–j). However, blocking ICAM-1 significantly reduced T cell-ASC adhesion to levels comparable to adhesion between non-activated T cells and ASCs (*p* = 0.045; Fig. 5a–d). Blocking ICAM-1 also resulted in a concurrent significant reduction of IFN-γ secretion (*p* = 0.002; Fig. 5e). These findings collectively suggest that ICAM-1 is important for mediating the adhesion between feline ASCs and T cell and may be involved in contact-dependent immunomodulation by feline ASCs.

## Discussion

Cats are increasingly used as translational models for MSC-based therapies, and a number of inflammatory feline diseases resemble human inflammatory conditions [32, 33]. Feline ASCs have been used in a number of clinical trials for diseases including feline chronic gingivostomatitis (FCGS), chronic enteropathy, chronic kidney disease, and feline asthma with varying degrees of success [3, 4, 34–36]. However, the exact mechanism(s) by which feline ASCs alter T cell responses remain vaguely understood. The objective of this study was to elucidate the underlying pathways utilized by feline ASCs to mitigate inflammatory conditions characterized by activated T cell proliferation.

MSCs can modulate T cell function, suppress T cell proliferation, and decrease T cell viability, but the mechanisms by which they accomplish these tasks are different between species and tissue sources. Based on our current study, we determined that feline ASCs inhibit T cell proliferation via cell cycle arrest in the G0–G1 phase, similar to murine BM-MSCs [37], equine BM- and cord blood-derived MSCs [38], and canine ASCs (unpublished data). MSCs from other tissues sources, including equine ASCs and cord tissue-derived MSCs inhibit T cell proliferation through induction of apoptosis [38]. Human MSCs cause T cell apoptosis through a pathway mediated by IDO and IL-10 [39, 40]. Despite the species and tissue source variation, mechanisms underlying MSC inhibition of T cell responses are mediated by soluble factors and/or direct cell-to-cell interactions.

The interaction of cell surface receptors and their respective ligands on target cells are crucial for cell communication and modulation of cell functions [41]. ICAM-1 is an inducible cell adhesion glycoprotein expressed on the surface of a wide variety of cell types, including MSCs across different species [42]. ICAM-1 interactions with the β2 integrin CD11a/CD18 (LFA-1) on the surface of lymphocytes are functionally important as costimulatory molecules for T cell activation [43]. In humans, ICAM-1 is constitutively expressed at a low level on the MSC surface but is significantly unregulated in the presence of pro-inflammatory cytokines, such as IFN-γ [44, 45]. Here we demonstrate that feline MSCs express ICAM-1 on their surface and that this molecule is similarly upregulated by activation. Further, our data demonstrate that this ligand plays a critical role in ASC-lymphocyte adhesion and signaling.

Although feline ASCs are capable of inhibiting lymphocyte proliferation in the absence of direct cell contact [8], the secretion profile of ASCs with and without direct cell-cell contact is very different. Our current data demonstrate that ICAM-1/LFA-1 interaction is critical for cell-cell adhesion and plays an important role in the secretion of immunomodulatory mediators, from both T cells (IFN-γ) and MSCs (PGE_2_), as blocking these ligands significantly reduced their concentration. Our findings mimic in vivo findings in a mouse model where blockade of ICAM-1 ligand also decreased IFN-γ secretion and reduced pulmonary barrier damage in T cell-induced acute lung injury [46]. In mice, it was also found that the overexpression of ICAM-1 on MSCs can enhance the immunosuppressive effects of MSCs, including modulating T cell responses, dendritic cell maturation, and secretion of immunomodulatory soluble factors in vitro [47].

We also evaluated the ligand pairs CD137-CD137L and PD-1/PDL-1. CD137 (4-1BB), an inducible protein expressed on both CD4+ and CD8+ T cells, is functionally involved in signaling T cell proliferation [48]. CD137-CD137L interaction has been implicated as one potential immunosuppressive mechanism used by human MSCs in the treatment of multiple sclerosis [49]. CD137-CD137L interaction has also been implicated for the paradoxical increase in IFN-γ that supports CD8 T regulatory expansion [50]. Similarly, programmed death-1 (PD-1) and its ligand, PD-L1, is an important inhibitory pathway of T cell response and has been implicated as a crucial interaction used by human MSCs to inhibit T cell responses [14, 51]. However, our data suggest that although CD137L and PDL-1 are expressed on activated feline ASCs, they are not the primary mediators of ASC T cell adhesion and do not mediate IFN-γ secretion in vitro.

Upon activation, feline ASCs secrete several immunomodulatory mediators, including IDO, PGE_2_, IL-6, IL-8, and TGF-β [8, 29, 52]. However, the principal immunomodulatory mediators used by MSCs appear to vary by species. Human MSCs primarily utilize IDO whereas canine MSCs, both bone marrow-derived and adipose-derived, utilize TGF-β and PGE_2_ to suppress lymphocyte proliferation [53–55]. With feline ASCs, we found that blocking IDO, with 1-methyltryptophan, or adding a TGF-β blocking antibody to the assay did not significantly alter T cell proliferation. Like dogs and horses, PGE_2_ is at least one soluble factor utilized by feline ASCs to block T cell proliferation as blocking PGE_2_ with indomethacin, a competitive inhibitor of PGE_2_, partially restored T cell proliferation [12, 53]. However, the role of PGE_2_ was modest compared to similar experiments conducted with equine ASCs [38], implying that other soluble factors are also likely involved in feline ASC-T cell interaction. Despite a trend, blocking both PGE_2_ and IDO did not significantly restore T cell proliferation likely due to small sample size and the variability of T cell responses to mitogens from different cat donors.

Our findings are in agreement with data from others that used a different PGE_2_ inhibitor, NS-398, to reverse the immunosuppressive effects of feline ASCs [52]. Although nitric oxide (iNOS) is implicated in the mechanism of MSC-mediated T cell suppression by both human and murine MSCs [28, 56], we found that feline ASCs do not produce a substantial amount of iNOS, either with or without activation. These findings correspond to a recent study where the level of iNOS RNA in feline ASCs was low or undetectable [29].

PGE_2_ is an eicosanoid lipid mediator which is produced by MSCs from most species, including human, murine, canine, equine, and feline [13, 57]. Although PGE_2_ may be pro-inflammatory in some contexts, it can also be immunosuppressive and is capable of decreasing IL-2 production from T cells and shifting CD4+ T cells from a predominantly cytotoxic Th1 response to a more balanced Th2/Th17-mediated response [58]. PGE_2_ also promotes the development of regulatory T cells and mediates their suppressive actions on effector T cells [59, 60]. These mechanisms may partially explain how feline ASCs are successfully used to treat FCGS, an immune-mediated inflammatory disease.

MSCs generally require licensing with IFN-γ to exert their immunosuppressive effects [61], our study showed that IFN-γ is produced by both CD4+ and CD8+ T lymphocytes upon mitogen activation and the production of IFN-γ from both T cell subsets is further enhanced by feline ASCs. These findings indicate that feline ASCs may be appropriate for therapeutic trials for both CD4+- and CD8+-mediated alloreactive diseases.

## Conclusion

Feline ASCs utilize PGE_2_ and ICAM-1/LFA-1 ligand interaction to inhibit T cell proliferation by causing cell cycle arrest in the G0–G1 phase. While many questions remained to be addressed, these findings provide a deeper understanding of the underlying mechanisms involved in the immunosuppression by feline ASCs and will lead to more efficient implementations of ASC-based therapy for the modulation of immune-mediated inflammatory disease models.

## Additional file

Additional file 1: **Figure S1.** Feline ASCs do not downregulate PD-1 expression on activated PBMCs. Flow analysis on PD-1 expression on activated T lymphocytes with and without co-incubation with feline ASCs. Representative image of flow cytometry analysis from 3 different MSC lines. (DOCX 185 kb)

## Data Availability

All datasets used and/or analyzed during the current study are available from the corresponding author on reasonable request.

## Abbreviations

ASC: Adipose-derived mesenchymal stem cell
BrdU: 5-Bromo-29-deoxyuridine
ConA: Concanavalin A
DPBS: Dulbecco’s phosphate buffered saline
ELISA: Enzyme-linked immunosorbent assay
FBS: Fetal bovine serum
IDO: Indoleamine 2,3 dioxygenase
IFN-γ: Interferon gamma
MLR: Mixed leukocyte reaction
MSC: Mesenchymal stem cell
PBMC: Peripheral blood monocular cell
PGE_2_: Prostaglandin E2
SPF: Specific pathogen free
TGF-β: Transforming growth factor beta
TNF-α: Tumor necrosis factor alpha
