# Supplementary material for: Mechanisms utilized by feline adipose-derived mesenchymal stem cells to inhibit T lymphocyte proliferation
Source: Stem Cell Res Ther. 2019 Jun 25;10:188. doi: 10.1186/s13287-019-1300-3 (PMC6593543; doi:10.1186/s13287-019-1300-3)
Supplement: Supplementary file 1 [file 13287_2019_1300_MOESM1_ESM.docx]

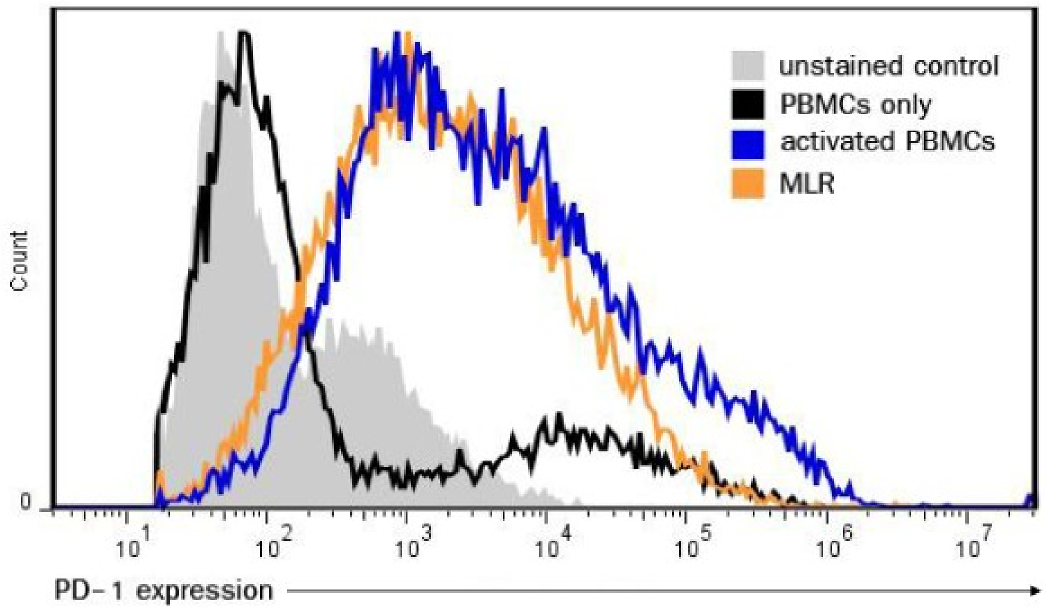

unstained control
PBMCs only
activated PBMCs
MLR
Count
0
10^1
10^2
10^3
10^4
10^5
10^6
10^7
PD-1 expression
